# Implantation of biomimetic polydopamine nanocomposite scaffold promotes optic nerve regeneration through modulating inhibitory microenvironment

**DOI:** 10.1186/s12951-024-02962-y

**Published:** 2024-11-07

**Authors:** Tonghe Pan, Yate Huang, Jinfei Wei, Chen Lai, Yangjun Chen, Kaihui Nan, Wencan Wu

**Affiliations:** 1 State Key Laboratory of Ophthalmology, Optometry and Vision Science, School of Ophthalmology & Optometry, Eye Hospital, Wenzhou Medical University, Wenzhou, 325027 Zhejiang China; 2 National Engineering Research Center of Ophthalmology and Optometry, Institute of Biomedical Engineering, Eye Hospital, Wenzhou Medical University, Wenzhou, 325027 Zhejiang China; 3 Shenzhen Key Laboratory of Human Tissue Regeneration and Repair, PKU-HKUST ShenZhen- HongKong Institution, Shenzhen, 518057 Guangdong China; 4 National Clinical Research Center for Ocular Diseases, Eye Hospital, Wenzhou Medical University, Wenzhou, 325027 Zhejiang China; 5 Oujiang Laboratory (Zhejiang Lab for Regenerative Medicine, Vision and Brain Health), Wenzhou, 325000 Zhejiang China

**Keywords:** Biomimetic scaffold, Reactive oxygen species, Polydopamine nanoparticles, Microenvironment modulation, Optic nerve regeneration

## Abstract

Optic nerve regeneration remains challenging worldwide due to the limited intrinsic regenerative capacity of retinal ganglion cells (RGCs) and the inhibitory microenvironment. Oxidative stress, induced by excessive reactive oxygen species (ROS) following optic nerve injury, is associated with prolonged neuroinflammation, resulting in a secondary injury of RGCs and the impairment of axon regeneration. Herein, we developed a bionic nanocomposite scaffold (GA@PDA) with immunoregulatory ability for enhanced optic nerve regeneration. The ice-templating method was employed to fabricate biopolymer-based scaffolds with a directional porous structure, mimicking the optic nerve, which effectively guided the oriented growth of neuronal cells. The incorporation of bioinspired polydopamine nanoparticles (PDA NPs) further confers excellent ROS scavenging ability, thereby modulating the phenotype transformation of microglia/macrophages from pro-inflammatory M1 to anti-inflammatory M2. In a rat optic nerve crush model, the implantation of GA@PDA scaffold enhanced survival of RGCs and promoted axonal regeneration. Our study offers novel insights and holds promising potential for the advancement of engineered biomaterials in facilitating optic nerve regeneration.

**Supplementary Information:**

The online version contains supplementary material available at 10.1186/s12951-024-02962-y.

## Introduction

Optic nerve, including retinal ganglion cells (RGCs) and their axons, is part of the central nervous system (CNS) and represents the sole means to transmit visual information from the eye to the brain [1]. Like most neurons in CNS, RGCs cannot spontaneously regenerate damaged axons when injured in adult mammals [2, 3]. Optic nerve injury-related ocular diseases, such as head trauma and glaucoma, generally lead to irreversible loss of vision. Despite the limited inherent regenerative capacity, environmental factors are considered as the other significant hurdle for optic nerve regeneration [1, 4]. Pioneering works by Aguayo et al. indicated that RGC axons could grow into a peripheral nerve graft [5], promoting both axon regeneration and RGC survival. Recently, Zeng et al. reported that decellularization, which effectively eliminates axon-inhibitory molecules such as myelin-associated glycoprotein and chondroitin sulfate proteoglycans, could optimize the inhibitory microenvironment and thereby facilitate neurite growth [6, 7]. These important discoveries suggested that regulating the neuroinhibitory microenvironment plays a key role in promoting optic nerve regeneration [8]. However, either peripheral nerve graft or decellularized optic nerve scaffold may present practical challenges for application due to potential issues such as immune rejection [9]. In this aspect, natural biopolymer-based scaffolds show great advantages benefiting from their easy availability, excellent biocompatibility and biodegradability, and low immunogenicity [10]. For example, sodium alginate and gelatin are widely used in neural tissue engineering, supporting cell growth without triggering immune responses. The scaffolds mimic the extracellular matrix (ECM) and offer tunable mechanical properties such as stiffness and porosity, creating an optimal environment for neural regeneration [11–13]. Despite the extensive utilization of scaffolds in tissue engineering [14–16], there still remains a paucity of research on biopolymer-based scaffolds for optic nerve regeneration.

Neuroinflammation is considered as one of the leading factors that impede the recovery of RGCs after primary optic nerve injury [17, 18]. As is well-accepted, resident microglia and migratory macrophages are key mediators of CNS inflammation [19]. Microglia/macrophages activation-induced inflammatory response is correlated with RGCs apoptosis [20–23]. Activated microglia/macrophages are basically divided into two extremes of status, i.e., pro-inflammatory M1 type and anti-inflammatory M2 type [24]. M2 phenotype of microglia/macrophages plays a critical role in inflammation inhibition, tissue repair, and neuroprotection [25–27]. Recent studies have shown that promoting the transition of microglia/macrophages from M1 phenotype to M2 phenotype successfully promoted RGCs survival after optic nerve crush (ONC) [28, 29] or retinal ischemia-reperfusion (RIR) injury [30]. However, they have focused on interventions targeting the RGC cell bodies in the retina, overlooking the primary lesion in the optic nerve. This is mainly due to the optic nerve’s deep location, the presence of the blood-brain barrier, and the severe disruption of axoplasmic flow and blood supply after injury, making localized treatment challenging. Since the axons of optic nerve are rich in mitochondria, they are more vulnerable to mitochondria dysfunction and oxidative stress [31]. The accumulation of reactive oxygen species (ROS) and subsequent induction of oxidative stress have been found in optic nerve injury-related ocular diseases like glaucoma and traumatic optic neuropathy (TON) [32, 33]. A cascade of inflammatory responses and oxidative stress during optic nerve injury could promote M1 polarization of microglia/macrophages, which leads to a more detrimental inhibitory microenvironment and ultimately results in the death of RGCs and loss of axons [34]. Therefore, the eradication of excessive ROS is crucial for the microglia/macrophages phenotypic transition towards the neuroprotective M2 subtype following optic nerve injury.

Polydopamine (PDA), a bioinspired synthetic analogue of naturally occurring melanin, has been widely used in biomedical areas due to its good biocompatibility and biodegradability and many other outstanding properties [35–42]. Benefiting from the abundant phenolic groups, PDA-based nanoparticles (PDA NPs) showed remarkable capability in scavenging ROS for the treatment of various diseases [43–47]. For example, PDA NPs were explored as a robust antioxidant to protect brain from injury in ischemic stroke through scavenging excess reactive oxygen and nitrogen species (RONS) [48–50]. Moreover, many studies indicated that anti-oxidative PDA-based biomaterials could regulate the injured microenvironment by promoting the polarization of microglia/macrophages from M1 to M2 [15]. Notably, PDA NPs have also been explored to treat several oxidative stress-related ophthalmic diseases such as age-related macular degeneration (AMD) [51–56]. Therefore, PDA NPs show great potential to serve as a microenvironment modulator for optic nerve regeneration.

**Fig. 1:**
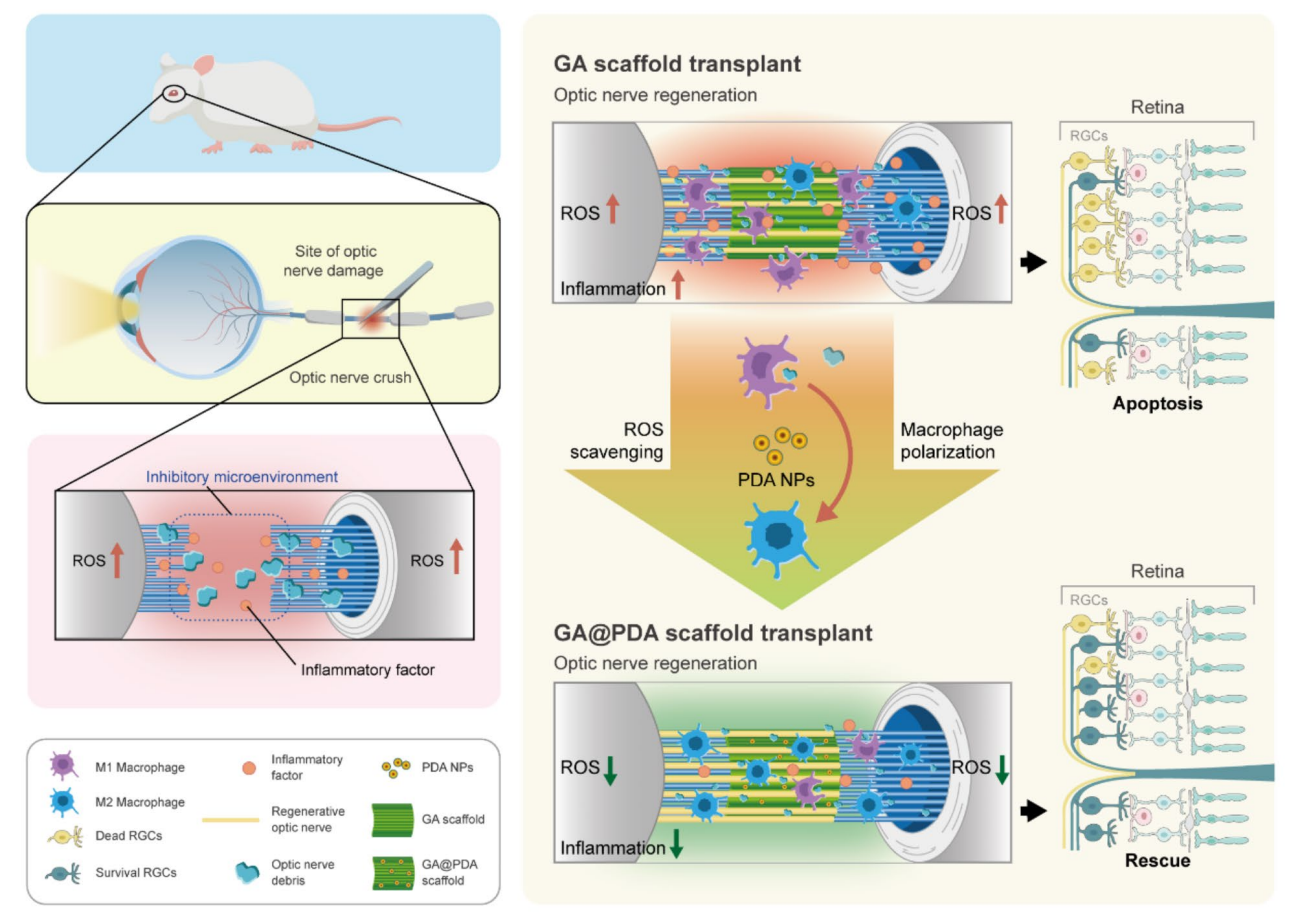
Schematic illustration of the therapeutic mechanism of ROS-scavenging biomimetic scaffold (GA@PDA) on RGCs protection and axonal regeneration through modulating inhibitory microenvironment after optic nerve injury

Herein, we fabricated a biopolymer-based scaffold (GA@PDA) with optic nerve-mimicking microstructure via ice-templating method [57]. Anti-oxidative PDA NPs were further loaded as a ROS-scavenger to modulate microglia/macrophage polarization. The biocompatibility, anti-oxidative and anti-inflammation properties of GA@PDA scaffold were well-assessed both in vitro and in vivo. The nanocomposite scaffold was supposed to down-regulate the level of oxidative stress, thereby inducing the transformation of microglia/macrophages from M1 to M2 (Fig. 1). As a result, the implantation of GA@PDA nanocomposite scaffold showed considerable therapeutic effect on promoting RGCs protection and axonal regeneration after optic nerve injury. Therefore, our study provides a novel microenvironment-regulating strategy for optic nerve regeneration via functional biopolymer scaffold-based tissue engineering.

## Materials and methods

### Chemicals and materials

Gelatin from porcine skin (Type A, gel strength ~ 300 g Bloom), dopamine hydrochloride, Live/Dead cell staining kit, and lipopolysaccharide (LPS) were purchased from Sigma-Aldrich (USA). Sodium alginate (M/G = 1:1) was purchased from Macklin (China). 2’,7’-Dichlorodihydrofluorescein diacetate (DCFH-DA) and Cholera Toxin Subunit B (CTB) were obtained from ThermoFisher Scientific (USA). Dulbecco’s Modified Eagle Medium/Nutrient Mixture F-12 (DMEM/F-12), trypsin, and fetal bovine serum (FBS) were purchased from Gibco (USA). All other solvents and reagents were used as received.

### **Synthesis of Polydopamine Nanoparticles** (**PDA NPs)**

PDA NPs were prepared based on previous reports [58, 59]. Briefly, ammonia aqueous solution (2 mL, NH_4_OH, ~ 28%) was mixed with ethanol (40 mL) and deionized water (DI water, 90 mL) at room temperature (RT) under stirring for 30 min. Afterwards, dopamine hydrochloride (0.5 g) in DI water (10 mL) was added slowly into the mixture. The reaction was continuously stirred at RT for 30 h. PDA NPs were obtained after centrifugation (11000–12000 rpm, 20 min) followed by washing with DI water for three times. The obtained PDA NPs were stored in 4℃ refrigerator for further use.

### Preparation of structurally biomimetic scaffolds

A structurally biomimetic gelation A/sodium alginate scaffold (denoted as GA) was fabricated using the directional freezing method [57]. Briefly, gelatin A (250 mg) was dissolved in DI water (10 mL) and stirred at 60℃ for 30 min, followed with the addition of sodium alginate under stirring for 30 min. Then, the mixture was cooled at RT and transferred to a homemade Teflon tube. The Teflon tube was placed in a temperature-gradient chamber designed by our laboratory, which maintained RT on the top and around − 60℃ at the bottom. After being frozen for 30 min, the Teflon tube was transferred to a freeze-drier and lyophilized for 48 h. Then, the scaffolds were collected from the Teflon tubes and further cross-linked using genipin (dissolved in 80% alcohol solution) for 24 h. The cross-linked scaffolds were soaked in the PDA NPs suspension (1 mg/mL) and softly shaken for 24 h at RT. Subsequently, the polydopamine nanocomposite scaffolds (GA@PDA) were obtained after lyophilization. Blank scaffolds (named as GA) were also fabricated without the addition of PDA NPs. The scaffolds were kept at 4℃ for further investigation.

### Characterizations of PDA NPs, GA and GA@PDA Scaffolds

The hydrodynamic diameter and size distribution of PDA NPs were determined by dynamic light scattering (DLS, Zetasizer Nano-ZS90, Malvern Instruments Limited, UK). The morphology of PDA NPs was observed by transmission electron microscope (TEM, FEI Tecnai F30, USA) and field emission scanning electron microscope (FE-SEM, Phenom Pharos, Thermo Fisher Scientific, USA). The structural morphology of optic nerve axon, GA and GA@PDA scaffolds was also observed by SEM. The samples were mounted on an aluminum stub using adhesive carbon tape and sputter-coated with gold in an argon atmosphere under low pressure using a Dynavac Mini Coater.

### Cell culture and cytotoxicity

Pheochromocytoma cells (PC12 cells) were a gift from the Institute of Advanced Materials for Nano-Bio Applications of Wenzhou Medical University. GA or GA@PDA scaffold was co-incubated with DMEM/F-12 culture medium at 37℃ for 3 days to obtain extracts, which were further used to evaluate the biocompatibility of scaffolds. Firstly, the cytotoxicity was evaluated by CCK-8 assay. Briefly, PC12 cells were cultured in DMEM/F-12 containing FBS (10%) in 96-well plates with a density of 8 × 10^3^ per well for 24 h. After discarding the culture medium, the cells were co-cultured with the extracts for 24, 48, and 72 h, respectively. Cell viability was measured by using Cell Counting Kit-8 (CCK-8, Dojindo, Japan). The cytocompatibility was further investigated by Live/Dead staining assay. PC12 cells were cultured in DMEM/F-12 containing FBS (10%) in 48-well plates with a density of 1 × 10^4^ cells per well for 24 h. The cells were then incubated with the extracts for 24, 48, and 72 h, respectively. After discarding the culture medium, cells were stained by Live/Dead assay, and then imaged using a fluorescent confocal microscope.

### Cell adhesion and directional growth on scaffolds

PC12 cells (5 × 10^4^ cells/mL) were seeded on GA or GA@PDA scaffolds separately. After 48 h, cells were washed gently three times with DPBS and the culture medium was replaced with fresh medium containing 0.1 µM Calcein-AM. Three-dimensional fluorescence images were obtained using a confocal microscope (LSM880, Carl Zeiss, Germany).

### Intracellular ROS determination

PC12 cells were seeded on coverslips in a 24-well plate with a density of 1 × 10^4^ per coverslip for 24 h. After the culture medium was removed, cells were incubated with DCFH-DA for 6 h at 37℃ in the dark. Cells were then washed with DPBS to remove excess DCFH-DA and incubated with DMEM/F12 containing FBS (10%) and Rosup (50 µg/mL). In the meantime, GA or GA@PDA scaffolds were placed in Trans-wells and put in the 24-well plate, followed by co-incubation for 1 h at 37ºC. Cells without any treatment and treated with only Rosup were defined as negative control and positive control, respectively. Fluorescence images were collected on the confocal imaging system.

### In Vitro Macrophage phenotype regulation

RAW264.7 cells were seeded on coverslips in a 24-well plate with a density of 3 × 10^4^ per coverslip for 24 h. Afterwards, the culture medium was replaced by DMEM containing LPS (1 µg/mL), LPS (1 µg/mL) + interleukin-4 (IL-4) (Abcam, 20 ng/mL), LPS (1 µg/mL) + GA, and LPS (1 µg/mL) + GA@PDA, respectively (*n* = 3). GA or GA@PDA scaffolds placed in Trans-wells were placed in the 24-well plate, followed by co-incubation for 24 h at 37ºC. Cells were stimulated with LPS to induce M1 polarization and stimulated with IL-4 to induce M2 polarization. The expression of M1 and M2 surface markers (CD86 and CD206) were detected by immunofluorescence to evaluate the M2 polarization induction ability of GA and GA@PDA scaffolds.

For flow cytometry, RAW264.7 cells were seeded into 6-well plates with a density of 5 × 10^4^ per well for 24 h. Afterwards, the culture medium was replaced by DMEM containing LPS (1 µg/mL), LPS (1 µg/mL) + IL-4 (20 ng/mL), LPS (1 µg/mL) + GA, and LPS (1 µg/mL) + GA@PDA, respectively. GA or GA@PDA scaffolds were placed in the 6-well plate via a Trans-well method. After co-incubation for 24 h, the RAW264.7 cells were collected into microtubes by Flow Cytometry Staining Buffer (Invitrogen, USA) and centrifuged. Flow antibodies for M1 and M2 surface staining include Anti-CD206-APC (1 µL per tube) and Anti-CD86-PE (5 µL per tube). All data were acquired on an Attune NxT flow cytometer (Invitrogen, USA), and analyzed with FlowJo.

### Immunofluorescence staining of cells

Cell-containing coverslips were washed with PBS three times and cells were fixed with 4% paraformaldehyde (PFA) for 30 min at RT, followed by incubation in permeabilization buffer (0.1% Triton X-100 in PBS) at RT for 1 h. Coverslips were washed with PBS three times and blocked with 1% BSA + 0.1% Triton X-100 in PBS for 1 h at RT. Then coverslips were incubated with TNF-α (1:300, Bioss), IL-1β (1:300, Proteintech), CD86 (Abcam, 1:300) or CD206 (Abcam, 1:300) primary antibodies for 12 h at 4ºC. Following washing three times with PBS, coverslips were incubated with secondary antibodies Cy3, Alexa Fluor488 (1:1000, Jackson ImmunoResearch) and DAPI (Beyotime) as a counterstained for 1 h at RT with gentle rocking in the dark. After being rinsed in PBS for three times and drying, coverslips were mounted with mountant (Beyotime). Image analysis was performed using a confocal microscope (LSM880, Carl Zeiss, Germany).

### Optic nerve crush (ONC) model and experimental procedures

The animal experimental protocols were approved by the Animal Care and Use Committee of Wenzhou Medical University (No.wydw2019-0137). Adult Sprague Dawley (SD) rats weighing 220–240 g were purchased from Jiesijie Experimental Animal Co., Ltd (Shanghai, China). All animals were housed in a temperature-controlled room subjected to a 12-hour light/12-hour dark cycle with free supply of food and water. All the rats were anesthetized with inhaled isoflurane for the surgery and 2.0% pentobarbital sodium for sacrifice.

The ONC animal model was performed according to our previous works [60–62]. Briefly, the optic nerve was exposed through a temporal, fornix-based conjunctival incision using blunt dissection. Subsequently, fine forceps (RWD, China) were employed to crush the nerve 2 mm behind the globe for a duration of 10 s. This procedure was performed to establish the crush group. The sham procedure involved the exposure of the optic nerve without inducing any crush injury, and this group was designated as the sham control. A piece of optic nerve with a length of ~ 2 mm was extruded to form a cavity after the sheaths of optic nerve were scratched using an insulin needle at 2–4 mm behind the globe. Subsequently, GA scaffolds (GA group) or GA@PDA scaffolds (GA@PDA group) were implanted into the optic nerve cavity. For observation of axon regeneration, CTB, a classic anterograde axon tracer, was injected intravitreally (1 µg/µL in 10 mM PBS, 5 µL) with a microinjector 2 days before the animal was sacrificed.

### Immunofluorescence labeling

After intracardiac perfusion with 4% PFA, the eye ball and optic nerve were removed, immersion-fixed with 4% PFA for 2 h and washed for 5 min in 10 mM PBS and immersed in 10% sucrose (Macklin, China) overnight. Tissues were embedded into Optimal Cutting Temperature compound (OCT, Tissue-Tek) and frozen at -80ºC overnight. Subsequently, 15-µm-thick sections were cut on a freezing microtome (CM 1950, Leica, Germany) and adhered onto glass slides. The sections were washed three times in 10 mM PBS, blocked with PBS containing 0.3% Triton X-100 and 10% normal goat serum at RT for 1 h, and incubated overnight with the antibodies, including rabbit polyclonal anti-TNF-α antibody (1:500, Bioss, bs-10802R), mouse monoclonal anti-IL-1β antibody (1:500, Proteintech, 66737-1-Ig), rabbit polyclonal anti-GAP-43 antibody (1:200, Abcam, ab75810), mouse monoclonal anti-βIII Tubulin antibody (1:500, Promega, G7121), mouse monoclonal anti-CD206 antibody (1:500, abcam, ab64693), mouse monoclonal anti-CD86 antibody (1:500, abcam, ab220188), Rabbit monoclonal anti-iNOS antibody (1:500, abcam, ab178945) and Rabbit polyconal anti- Arg-1 antibody (1:500, proteintech, 16001-1-AP). Slides were washed three times in PBS and incubated in the appropriate secondary antibodies CyTM3 or Alexa FluorTM488 (1:1000, Jackson Immuno Research) for 2 h at RT. After being washed with PBS, slides were covered using antifade mounting medium with DAPI (Beyotime). Fluorescence images were obtained using a confocal microscope (LSM880, Carl Zeiss, Germany) or fluorescence microscope (DM4B, Leica, Germany).

### Quantification of RGCs and regrown axons

After the animals were sacrificed, the retinas were collected and subjected to immunofluorescent staining for the purpose of quantifying RGCs. Flat-mounted retinas were divided into 4 parts and examined under a fluorescence microscope (DM4B, Leica, Germany). Two selected areas at 2 and 4 mm from the optic disc were photographed in the four retinal quadrants with the magnification of ×200. The βIII Tubulin labeled RGCs in the photographs were counted using the ImageJ, and the density of labeled RGCs per mm^2^ was calculated by averaging the 8 counts in each retina.

The number of CTB labeled axons crossing a line at distance *d* (0.5, 1, 1.5–2.0 mm) from the end of lesion site was counted [63].

$$\sum {{a_d} = \pi {r^2}} \times \left( {average{\text{ }}axons/mm{\text{ }}width} \right)/t$$

### Western blotting analysis

Rats were sacrificed at 3-week after modeling with 2.0% pentobarbital sodium (0.3 mL/100 g) and retinas were collected into cold PBS with the ice-bath. The tissues were then homogenized in RIPA lysis buffer containing 1% protease inhibitor cocktail and 1% phosphatase inhibitor cocktail, and broken up with an ultra-sonication machine with ice-bath for 30 min. After centrifugation with a speed of 14,000 rpm for 10 min at 4ºC, supernatants were collected and detected using Micro BCA Protein Assay Kit (23235, Thermo scientific, USA). For SDS-polyacrylamide gel electrophoresis process, the proteins were transferred onto the polyvinylidene difluoride (PVDF) membrane and blocked with blocking buffer (Epizyme) for 30 min. After being washed three times with Tris-Buffered Saline with Tween (TBST), the PVDF membranes were cut to the right size and incubated with primary antibodies as above mentioned overnight at 4ºC. After washing with TBST three times for 5 min each time, membranes were incubated with Goat anti-rabbit IgG (H + L) IRDye 800CW (1:8000, Odyssey) and Goat anti-mouse IgG (H + L) IRDye 680LT (1:8000, Odyssey). The images were acquired and analysed by Odyssey CLx Image Studio.

### Statistical analysis

All data were expressed as the mean ± standard deviation (SD). All statistical charts and analyses were carried out using GraphPad Prism (Version 8.02). Significant differences between the groups were determined by one-way analysis of variance (ANOVA) at confidence levels of 95% (*, *P* < 0.05), 99% (**, *P* < 0.01) and 99.9% (***, *P* < 0.001).

## Results and discussion

### Characterization of PDA NPs and biomimetic scaffolds

**Fig. 2:**
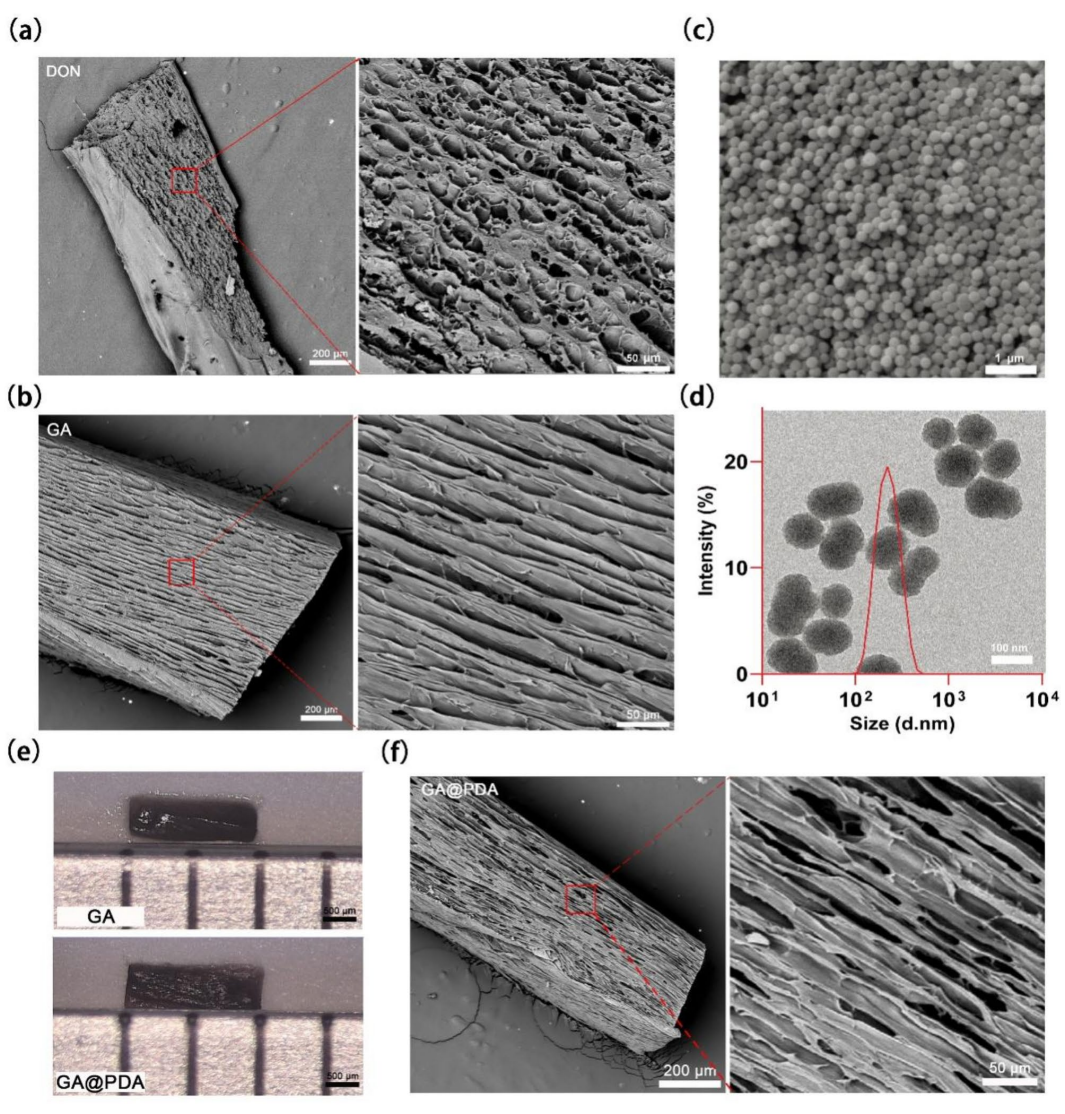
SEM images of longitudinal section of **(a)** decellularized optic nerve (DON) and **(b)** GA scaffold. **(c)** SEM image of PDA NPs. **(d)** TEM image of PDA NPs and the size distribution of PDA NPs by DLS. **(e)** Macroscopic views of GA and GA@PDA scaffolds. **(f)** SEM images of longitudinal section of GA@PDA scaffold

Biopolymer-based scaffolds with mechanical properties mimicking the ECM have been extensively developed to enhance neural regeneration [64]. The successful application of these scaffolds in CNS repair, such as spinal cord injury, has greatly stimulated our exploration of their potential in the field of optic nerve regeneration. Moreover, optic nerve axons are usually oriented longitudinally, therefore, scaffolds with oriented microstructures could be advantageous to guide and promote the extension and growth of axon fibers in a particular direction [65]. In this study, we fabricated biopolymer (gelatin and alginate)-based GA scaffolds with varying ratios of gelatin and sodium alginate through directional ice-templating (gradient freezing) method, with gelatin concentrations ranging from 25 mg/mL to 200 mg/mL and sodium alginate concentrations from 1 mg/mL to 10 mg/mL. The cross-sectional and longitudinal morphologies of these scaffolds were observed using SEM (Figure S1). The GA scaffold (G25A10) containing 25 mg/mL gelatin and 10 mg/mL alginate was selected for further investigation due to its more regular porous microstructure and similar oriented microchannels with that of decellularized optic nerve (DON) (Fig. 2a). The microchannel diameter of GA scaffold (23.8 ± 3.4 μm) was close to that of DON (21.0 ± 4.1 μm) (Fig. 2a, b), which can be advantageous for promoting axon regeneration axially. The scaffolds were further cross-linked with genipin, a well-considered safer cross-linker than glutaraldehyde, to improve the mechanical property and stability. The as-prepared scaffolds were found to be degraded progressively after immersing in PBS for 8 weeks (Figure S2). Furthermore, the compressive modulus of the cross-linked GA scaffold was measured to be 33.92 kPa, indicating that it has sufficient compressive resistance to prevent the collapse of the optic nerve dura mater and provide an adequate space for axon regeneration. (Figure S3) [16].

PDA NPs, which can be employed as an efficient ROS scavenger, were synthesized using a self-polymerization method. The successful preparation of uniform PDA NPs with spherical morphology was confirmed by both SEM and TEM observation (Fig. 2c, d). The hydrodynamic size of PDA NPs was measured by DLS to be 182.8 ± 37.3 nm with a low polydispersity index (PDI) value of ~ 0.05, indicating the monodisperse property of PDA NPs. Afterwards, PDA NPs were loaded into GA scaffolds to obtain nanocomposite GA@PDA scaffolds for the purpose of endowing them with ROS-scavenging capability. The successful encapsulation of PDA NPs was verified by SEM observation (Figure S4). The GA and GA@PDA scaffolds used for implantation were displayed in Fig. 2e, showing similar macroscopic appearance with a dark brown color, due to genipin cross-linking [66]. Moreover, the incorporation of PDA NPs exhibited negligible impact on the oriented microchannel microstructure of GA scaffolds, as shown by SEM (Fig. 2f).

### Biocompatibility and directionally guided cell growth

**Fig. 3:**
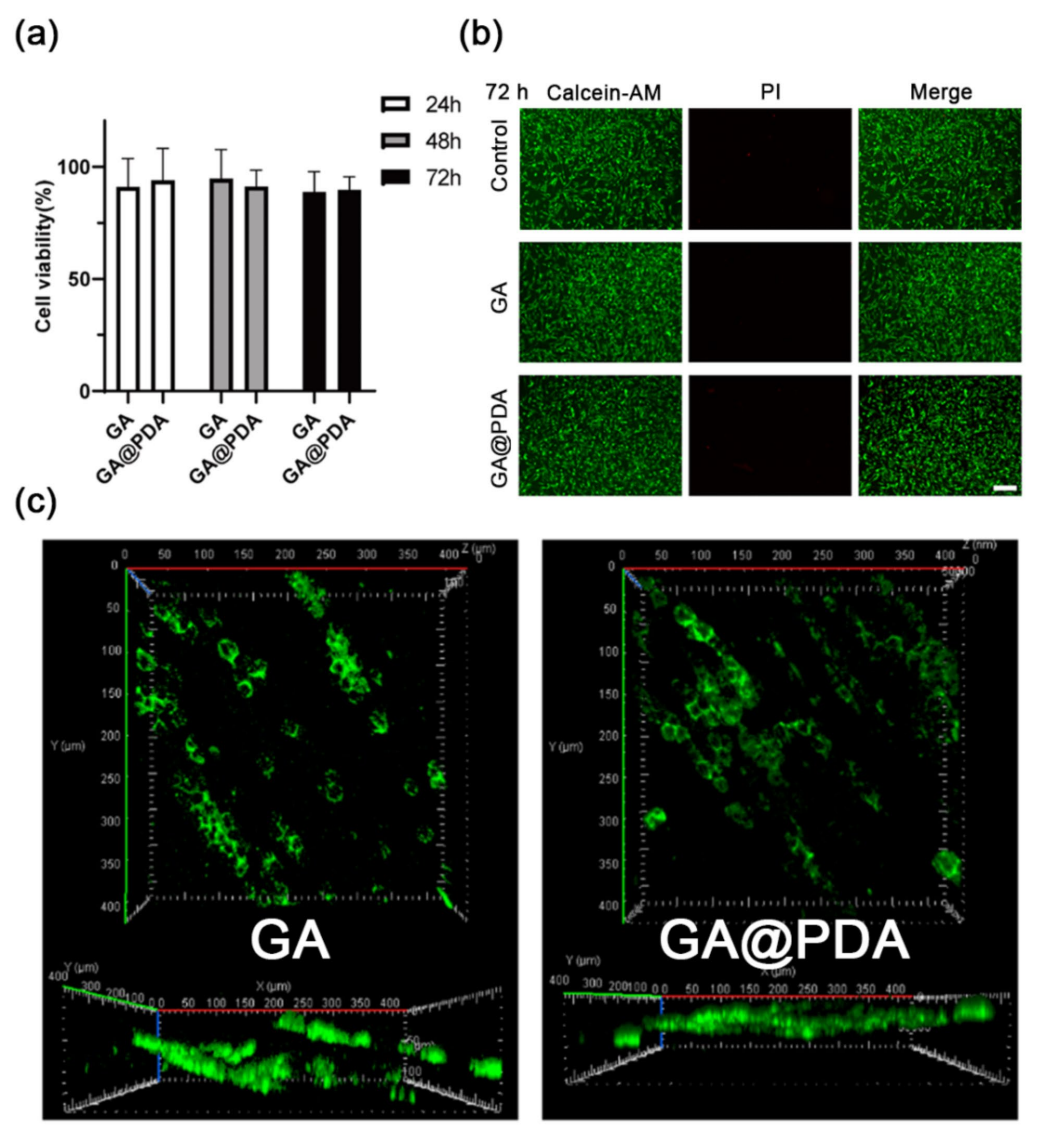
**(a)** Cell viability of PC12 cells after co-incubation with the extracts of GA or GA@PDA scaffold for 24, 48, and 72 h. **(b)** Dual-stained fluorescence images of PC12 cells incubated with the extracts of GA@PDA scaffold for 72 h via live/dead staining assay. Scale bar = 100 μm (*n* = 5). **(c)** 3D images of PC12 cells grew on GA or GA@PDA scaffolds

Good biocompatibility is a prerequisite for the use of scaffolds in tissue engineering. The PC12 neuronal cell line, which offers an extensively used model in neurobiology, was adopted as an alternative to RGCs for evaluating the in vitro biocompatibility of GA and GA@PDA scaffolds. As shown in Fig. 3a, over 85% of cell viability was retained after co-incubating PC12 cells with extracts of GA or GA@PDA scaffolds for 24, 48, and 72 h. The biocompatibility of the scaffolds was also verified by Live/Dead assay based on Calcein AM (green means live cells) and propidium iodide (red means dead cells) staining. As we can see, the majority of cells were stained green, and dead cells were negligible after culturing for 24, 48, and 72 h (Fig. 3b, S5). The excellent cytocompatibility should be attributed to the favorable biosafety of natural gelatin and alginate biopolymers.

PC12 cells were further cultured on GA or GA@PDA scaffolds and stained using Calcein AM to visualize the directional growth possibility. Benefiting from the oriented microchannel structure, PC12 cells were found to directionally migrate along the microchannels of both GA and GA@PDA scaffolds (Fig. 3c). This result demonstrated that the scaffolds cross-linked by genipin could maintain their oriented microchannel structure during cell culture, thus exhibiting great potential for guiding directional regeneration of optic nerve axons.

### In vitro ROS-scavenging and immunoregulation properties

**Fig. 4:**
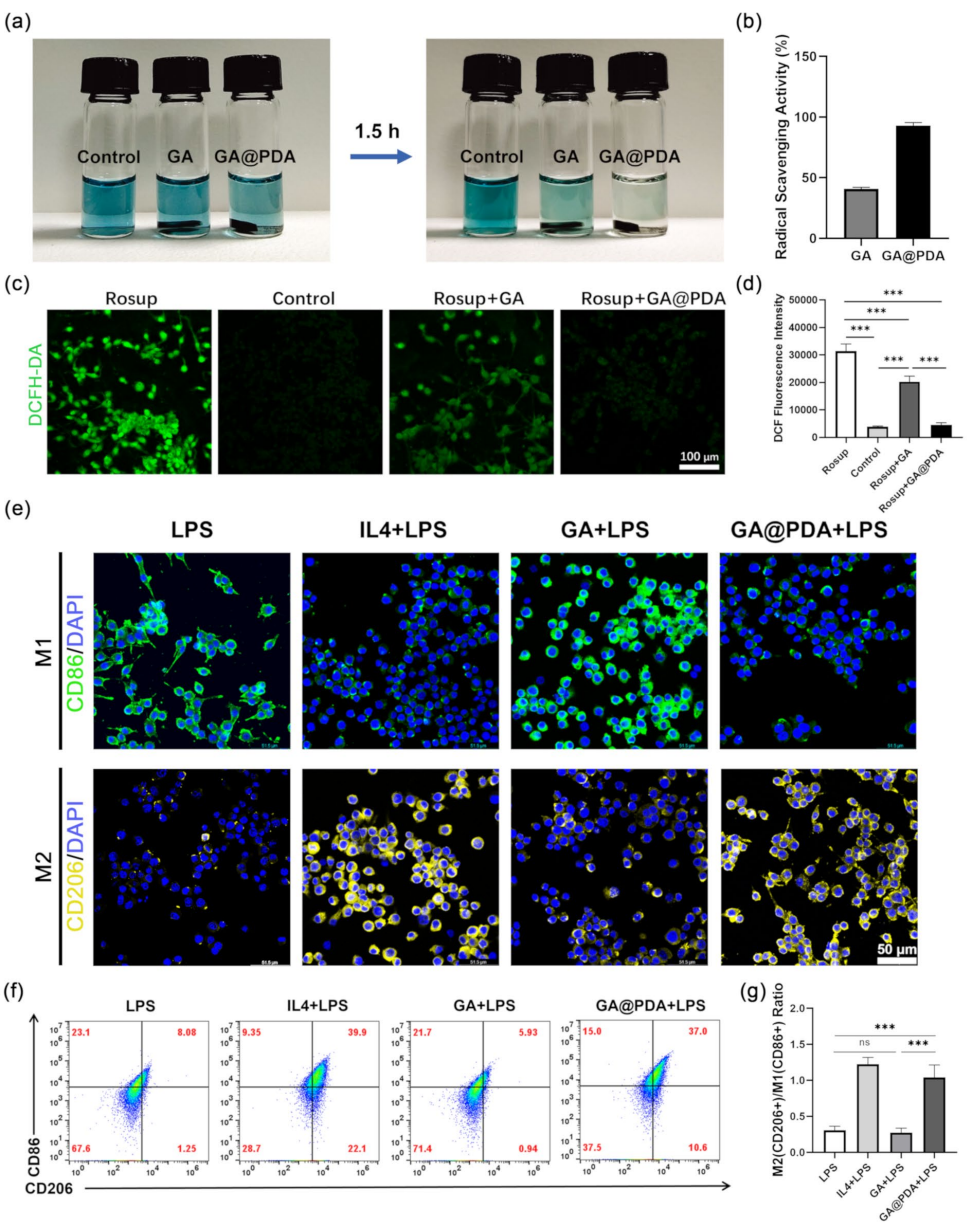
Evaluation of anti-oxidative and immune-regulating effects of GA and GA@PDA scaffolds. **(a)** The color change of ABTS⁺^·^ solution after co-incubation with GA and GA@PDA scaffolds. **(b)** ABTS⁺^·^ scavenging rate of the scaffolds. **(c)** DCF fluorescence images and **(d)** mean fluorescence intensity of PC12 cells upon various treatments (*n* = 5). **(e)** CD86 and CD206 staining images of RAW264.7 cells upon various treatments. **(f)** Representative flow plots of M1 (CD86^+^) and M2 (CD206^+^) of RAW264.7 cells upon various treatments. **(g)** Quantitative data of M2 (CD206^+^)/M1 (CD86^+^) ratio after different treatment (*n* = 3). ***p < 0.001, and ns, no significance

Total Antioxidant Capacity Assay Kit with ABTS was utilized to assess the anti-oxidative capacity of GA and GA@PDA scaffold. The color of ABTS⁺^·^ solution changed from blue to colorless when GA@PDA scaffold was immersed into ABTS⁺^·^ solution for 1.5 h, indicating nearly complete clearance of ABTS⁺ radicals (Fig. 4a). The absorbance values at 735 nm were used to calculate the free radical scavenging rate, which was 40.8 ± 1.4% and 93.1 ± 2.4% for GA and GA@PDA scaffold (Fig. 4b), respectively. The excellent ABTS⁺^·^ scavenging ability of GA@PDA scaffold should be ascribed to the loaded PDA NPs, which were widely considered as an excellent ROS scavenger. GA scaffold also showed some extent of anti-oxidative capability, probably due to the free radical scavenging ability of hydroxyl or phenolic hydroxyl groups of alginate and gelatin [67, 68].

Afterwards, the intracellular ROS scavenging capability of GA and GA@PDA scaffolds was investigated by using reactive Oxygen Species Assay (DCFH-DA). After treatment with Rosup, a robust green DCF fluorescence was observed in PC12 cells, indicating a significant increase in intracellular ROS levels (Fig. 4c). Interestingly, co-incubation with GA or GA@PDA scaffolds could remarkably decrease the ROS level due to their good ROS-scavenging ability as aforementioned (Fig. 4d). Moreover, GA@PDA scaffold exhibited significantly enhanced efficacy in scavenging ROS compared to GA scaffold, which was in accordance with the ABTS⁺^·^ scavenging result.

Oxidative stress plays an important role in activating the immune response following optic nerve injury. Overactivation of microglia/macrophages is considered to be correlated with RGCs apoptosis and axon degeneration [28, 69]. PDA NPs, a well-known free radical scavenger, have been reported to modulate inflammatory microenvironment and to promote M2 polarization of microglia/macrophages [43]. Therefore, the immune-regulating capability of GA@PDA scaffold was firstly investigated in vitro by using RAW264.7 cells (Fig. 4e) [70–72].

Immunofluorescence staining was utilized to visualize CD206 (a marker for M2) and CD86 (a marker for M1) expression. LPS was used to induce M1 phenotype of RAW264.7 cells as confirmed by strong CD86 fluorescence staining. Interestingly, the treatment with GA@PDA scaffold led to a significant enhancement in the fluorescence intensity of CD206 staining, accompanied by a notable reduction in the fluorescence intensity of CD86 staining, indicating successful polarization of LPS-induced M1 phenotype of RAW264.7 cells towards an M2 phenotype. In contrast, the GA + LPS group exhibited weak CD206 staining in the cells, potentially due to its limited anti-oxidative ability. Flow cytometry was further performed to quantify the proportion of M1 and M2 polarization of RAW264.7 cells (Fig. 4f). The proportion of M2 macrophages (CD206^+^) in the IL-4 + LPS and GA@PDA + LPS groups were significantly higher than that in the LPS and GA + LPS groups (Fig. 4g), indicating that GA@PDA effectively promoted the transformation of M1 macrophages stimulated by LPS into M2 phenotype, similar to IL-4.

### GA@PDA scaffold promotes RGCs survival and axonal regeneration

**Fig. 5:**
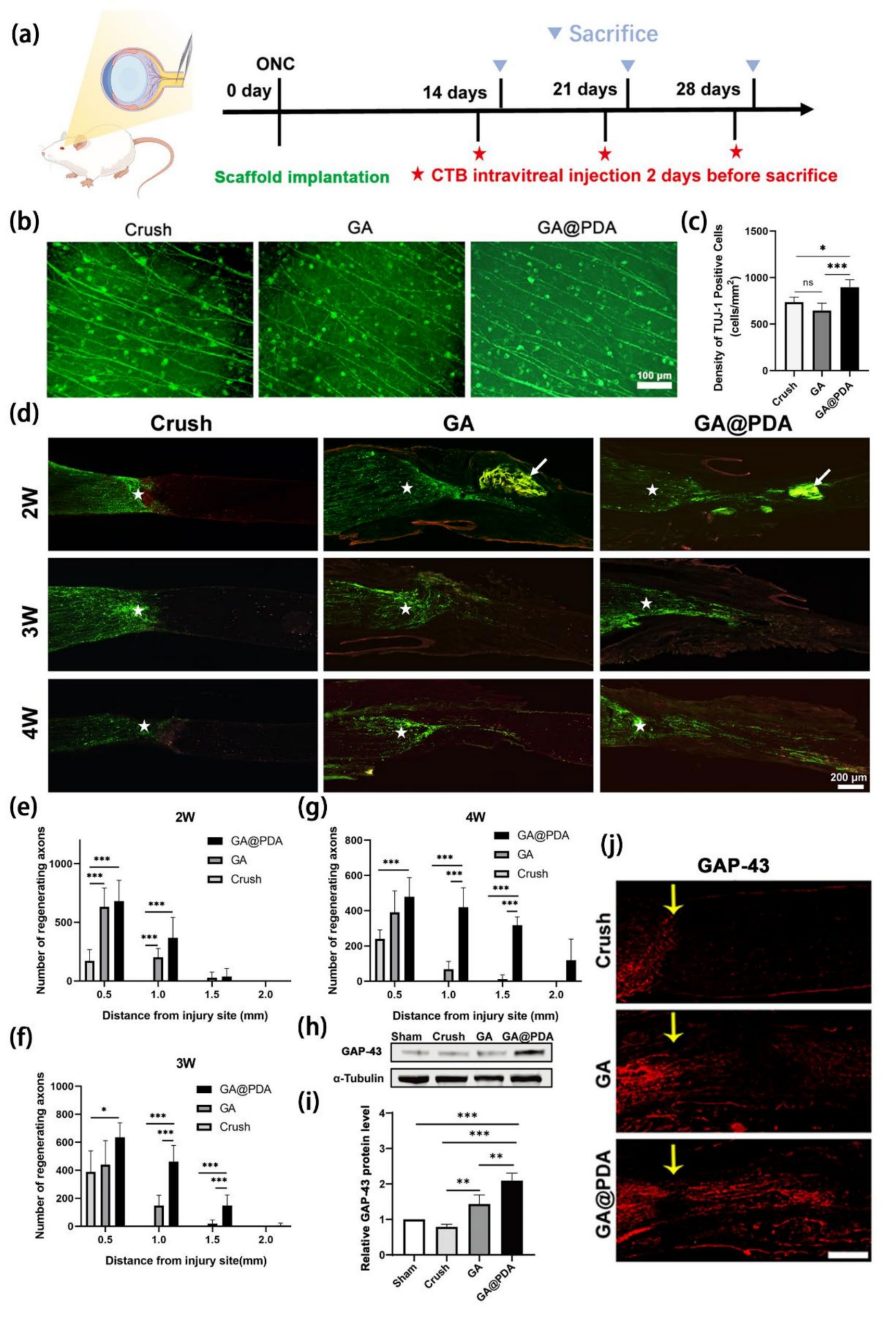
Implantation of GA@PDA scaffold protects RGCs and promotes axon regeneration after ONC. **(a)** Flow chart of animal experiments. **(b)** Immunofluorescence staining images of retinas labeled with βIII-tubulin at Week 3 after ONC and subsequent treatments. **(c)** Quantitation of survival RGCs after 3 weeks of scaffold implantation (*n* = 5). **(d)** Fluorescence images of optic nerve axons labeled with CTB (green) after 2 weeks, 3 weeks and 4 weeks of scaffold implantation. White asterisks indicate the ONC site, and white arrows denote the implanted scaffold. **(e-g)** Quantitation of axon regeneration at 2, 3, and 4 weeks post scaffold implantation (*n* = 5). The lesion site is marked with white asterisks. The white arrows indicate the implanted scaffolds. **(h)** Western blot analysis and **(i)** expression quantification of GAP-43 in each group (*n* = 3). **(j)** Immunofluorescence staining images of optic nerve axons labeled with GAP-43 (red) at Week 3 after different treatments. Yellow arrows indicate the ONC site. *p < 0.05, **p < 0.01, ***p < 0.001, and ns, no significance

To evaluate the in vivo function of GA and GA@PDA scaffolds, an optic nerve crush rat model was established. The surgical images for each group are shown in Figure S6. The sheath of optic nerve was longitudinally opened and 2 mm of the crushed optic nerve was removed. As for the GA or GA@PDA group, 2 mm long GA or GA@PDA scaffold was carefully fitted into the gap of optic nerve. The animals were sacrificed at week 2, week 3, and week 4 (Fig. 5a). The whole optic nerve was sampled and fixed at different time intervals. As shown in Figure S7, the implanted scaffolds were found to degrade progressively, which should be attributed to the good biodegradability of gelatin and alginate.

To evaluate the neuroprotective effect of GA@PDA, the number of survival RGCs was compared. βIII-Tubulin (TUJ-1) was used to label neuronal cells, particularly RGCs. Although TUJ-1 can also label other neuronal cells in the retina, such as bipolar and horizontal cells, it is primarily used to assess the survival rate of RGCs[60, 61]. The number of TUJ-1 positive cells per mm^2^ was calculated at the 3-week time point following ONC and subsequent treatments. As shown in Fig. 5b, more TUJ-1 positive cells were found in the GA@PDA group after ONC as compared to the Crush and GA groups. The density of RGCs was quantified as 737 ± 53 cells/mm² in Crush group, 647 ± 78 cells/mm² in GA group, and 895 ± 84 cells/mm² in GA@PDA group (Fig. 5c), thereby demonstrating the neuroprotective efficacy of GA@PDA scaffold implantation. We found that GA@PDA group did not significantly increase the survival rate of RGCs, even though there was a certain statistical difference. This is because RGCs face a very complex and diverse death mechanisms after optic nerve injury, including ferroptosis, apoptosis, necrosis, pyroptosis, and so on. A single inflammatory pathway may not be able to comprehensively inhibit the loss of RGCs.

The neurogenesis effect of GA@PDA scaffolds was further studied by using CTB, a classic anterograde axon tracer. As shown in Fig. 5d, there were very limited new axons in the Crush group even after 4 weeks. By contrast, both GA and GA@PDA scaffold implantation realized obvious axon regeneration from the lesion site (marked as asterisks). Moreover, the length and density of the regrown axons increased with time, and moderate fluorescence signals of CTB could be observed even at 2 mm (Week 4) beyond the crush site in GA@PDA group. The quantification of regenerated axons at various distances from the injury site during different time intervals (2, 3, and 4 weeks) was depicted in Fig. 5e-g. Compared with the other groups, significant increase in both length and density of regenerated axons was achieved for GA@PDA group, indicating that the implantation of GA@PDA scaffold could promote optic nerve regeneration following ONC. Enhanced axonal regeneration was also observed in the GA group due to the removal of the inhibitory factors (i.e., myelin debris), which released the intrinsic axonal regeneration capacity of RGCs, as reported in our previous studies [4, 61]. However, RGCs still suffered from inflammation and apoptosis due to the limited antioxidant capability of GA scaffold. In contrast, GA@PDA scaffold with enhanced anti-oxidative property exhibited superior regulation of the microenvironment, thereby promoting both RGC survival and axonal regeneration, thereby resulting in a higher density of axons.

To further verify whether the implanted scaffold activated the regeneration program of optic nerve axons, the expression level of growth associated protein-43 (GAP-43) was investigated via Western blot and immunofluorescence. The Western blot results revealed a significant increase in the level of GAP-43 for both GA and GA@PDA groups, with the latter demonstrating the most pronounced elevation of GAP-43 expression (Fig. 5h, i). As shown in Fig. 5j, GAP-43 labeled axons were barely found beyond the injured site (yellow arrow) for Crush group, in good accordance with the CTB result in Fig. 5c. In comparison, obvious GAP-43 fluorescence could be found to pass through the injured site after scaffold implantation. The Western blot results also revealed a significant increase in the level of GAP-43 for both GA and GA@PDA groups, with the latter demonstrating the most pronounced elevation of GAP-43 expression (Fig. 5i-j), which is consistent with the above Western blot result. Together, the expression of GAP-43 was upregulated in the injury site after scaffold implantation, further confirming the neurogenesis effect of GA@PDA scaffold.

### Implantation of GA@PDA scaffold alleviates oxidative stress and down-regulates inflammation levels

**Fig. 6:**
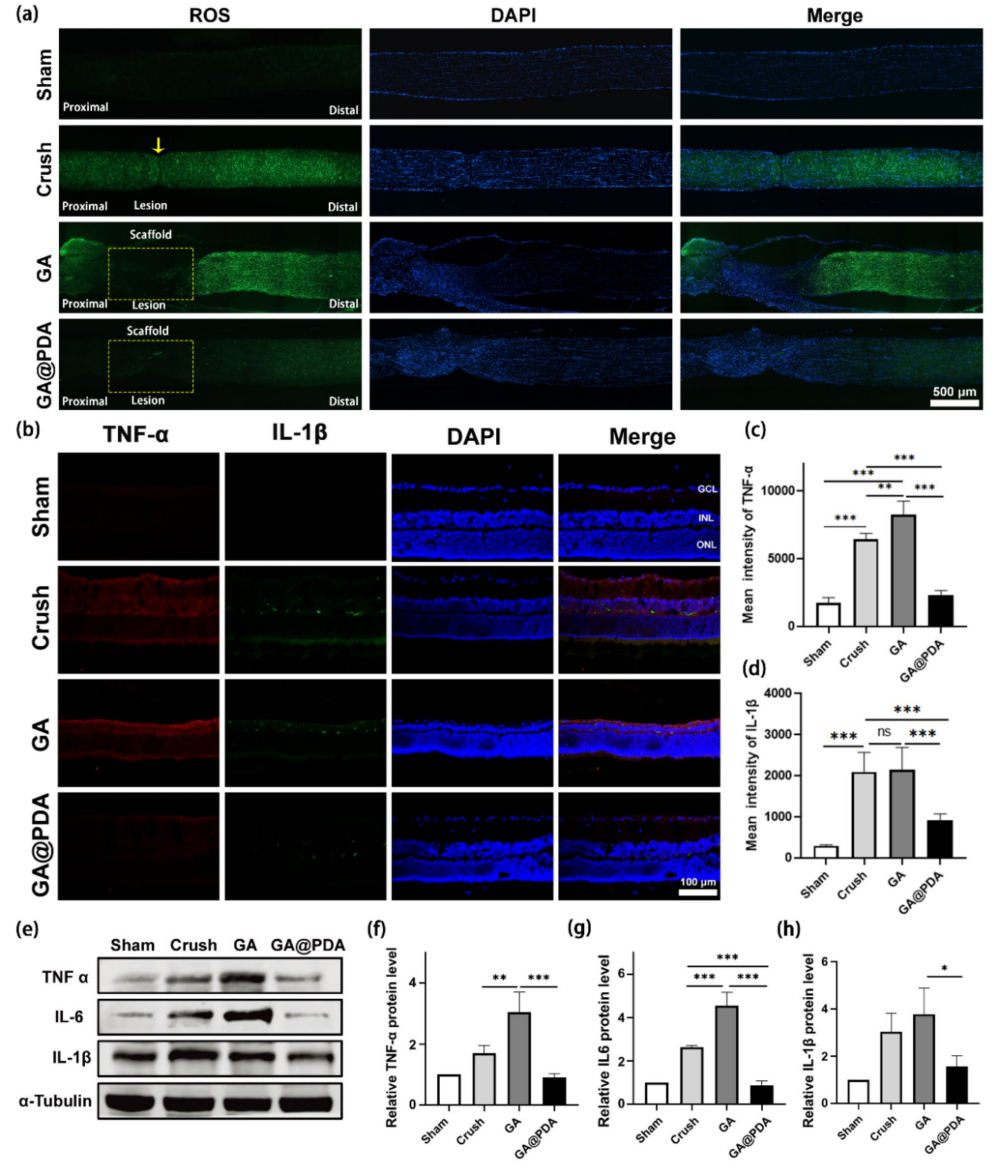
Implantation of GA@PDA scaffold scavenged the accumulated ROS and down-regulated inflammation after ONC. **(a)** Fluorescence images of ROS in the optic nerve labeled with the fluorescence probe DCF (green) 3 weeks post scaffold implantation. DAPI (blue) was used to stain nuclei. **(b-d)** Immunofluorescence staining images and mean fluorescence intensity of IL-1β and TNF-α in the retina 3 weeks post scaffold implantation (*n* = 5). **(e-h)** Western blot analysis of TNF-α, IL-1β and IL-6 in each group (*n* = 3). *p < 0.05, **p < 0.01, ***p < 0.001, and ns, no significance

As is known, the optic nerve axon is rich in mitochondria, and mitochondria are recognized as the main source of ROS [73]. As shown in Fig. 6a, obvious accumulation of ROS (marked as green) was found in the entire optic nerve axon after ONC (Crush group). The implantation of GA scaffold demonstrated limited efficacy in ROS elimination, likely attributed to its constrained antioxidant capacity. However, the ROS level decreased dramatically after the implantation of GA@PDA scaffold. The excellent ROS scavenging effect should be ascribed to the incorporation of PDA NPs, which have been extensively employed for treating oxidative stress-related diseases [43, 46]. Therefore, the addition of PDA NPs plays a critical role in reducing the accumulation of ROS in the optic nerve after ONC. Furthermore, we also found that the accumulation of ROS in the distal optic nerve is significantly higher than it in the proximal segment during fluorescence staining. This is because both axoplasmic flow and blood flow are blocked after optic nerve injury. The proximal segment, still connected to the cell body, retains a compensatory ability. While the distal segment, severed from the cell body, suffers from insufficient nutrient and oxygen supply. This deficiency causes ROS to accumulate in mitochondria, leading to significant disparities in ROS levels. This phenomenon may contribute to the challenges of axonal regeneration across the distal optic nerve.

It is widely acknowledged that oxidative stress is intricately associated with inflammation and plays a pivotal role in numerous neurodegenerative disorders [74, 75]. It should be emphasized that intraocular inflammation is a double-edged sword. While appropriate intraocular inflammation has been reported to facilitate axon regeneration [76], excessive retinal inflammation, especially chronic neuroinflammation, can induce the M1 polarization of microglia/macrophages, leading to a more detrimental inhibitory microenvironment and subsequent apoptosis of RGCs [77]. Based on this, we analyzed the inflammatory factors (TNF-α, IL-1β and IL-6) in the retina where RGC soma locates by both immunofluorescence staining and Western blotting after ONC and different treatments. At Week 3 post ONC, obvious inflammation, which mainly existed in ganglion cell layer (GCL) and inner nuclear layer (INL), was found in the retina for Crush group (Fig. 6b-d). Compared to the Crush group, the GA group exhibited more pronounced atrophy in each layer, accompanied by diffuse chronic inflammation observed in both the GCL and INL. Furthermore, the expression levels of IL-6 and TNF-α were found to be elevated even beyond those observed in the Crush group. By contrast, significant down-regulation of inflammatory cytokines was observed in the GA@PDA group, which was further confirmed by Western blot results shown in Fig. 6e-h. The anti-inflammatory effect of GA@PDA scaffold could be attributed to the incorporated PDA NPs, which have demonstrated remarkable efficacy in mitigating oxidative stress in vivo.

### GA@PDA scaffold modulates M2 polarization of retinal microglia/macrophages

**Fig. 7:**
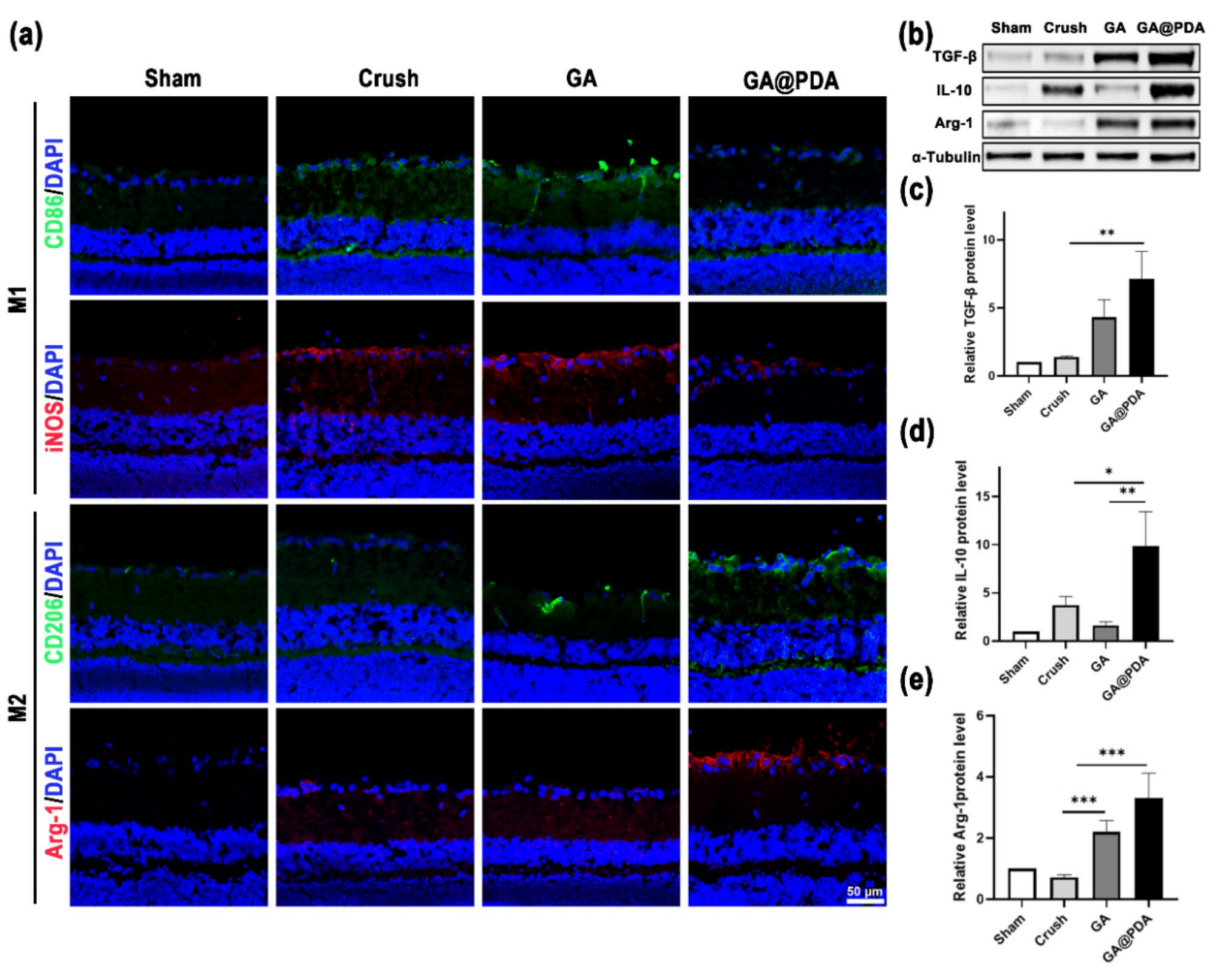
GA@PDA scaffold implantation regulates phenotypic transformation of retinal microglia/macrophages after ONC. **(a)** Immunofluorescence staining images of CD86, iNOS (M1) and CD206, Arg-1 (M2) in retina after 3 weeks of scaffold implantation (*n* = 5). **(b-e)** Western blot analysis of TGF-β, IL-10, and Arg-1 in each group (*n* = 3). *p < 0.05, **p < 0.01, and ***p < 0.001

It is generally recognized that neuroinflammation plays a key role in the pathological process of neurodegenerative diseases [74]. After ONC, overactivated microglia/macrophages are prone to polarize towards M1 phenotype in response to injury stimulation and oxidative stress [18]. This polarization leads to an inflammatory cascade reaction and a deteriorated inhibitory microenvironment, ultimately resulting in impaired axon regeneration and apoptosis of RGCs. It has been reported that suppressing the excessive activation of microglia/macrophages can alleviate neuroinflammation and contribute to RGCs protection and optic nerve regeneration [78]. We have already testified the immunoregulation capability of GA@PDA scaffold in vitro, here the in vivo effect of GA@PDA scaffold on M2 polarization of retinal microglia/macrophages was further investigated by both immunofluorescence staining and Western blot analysis. As shown in Fig. 7a, the fluorescence signal of M1 microglia/macrophages (CD86^+^ and iNOS^+^) was significantly up-regulated in the GCL layer in the Crush and GA groups, indicating that considerable microglia/macrophages were polarized to M1 phenotype after ONC. In comparison, the implantation of GA@PDA scaffold significantly decreased the proportion of M1 microglia/macrophages, while markedly enhancing the infiltration and distribution of M2 microglia/macrophages (CD206^+^ and Arg-1^+^). Western blot analysis was subsequently conducted to quantify the expression levels of pivotal proteins associated with M2 microglia/macrophages, including TGF-β, IL-10, and Arg-1. In accordance with the above immunofluorescence staining results, the biomarkers of M2 microglia/macrophages (TGF-β, Arg-1) and inflammatory inhibitory factor (IL-10) were significantly up-regulated for GA@PDA group (Fig. 7b-e). Therefore, it could be inferred that the implantation of GA@PDA scaffold effectively modulates the inhibitory microenvironment following ONC by inducing M2 polarization of microglia/macrophages, thereby enhancing RGCs survival and promoting axonal regeneration. The immunomodulatory ability can be attributed to the incorporation of antioxidant PDA NPs, which are widely utilized as a potent ROS scavenger and a facilitator of M2 macrophage polarization [15].

## Conclusions

In summary, we reported a nanocomposite scaffold which achieved favorable efficacy in promoting optic nerve regeneration via microenvironment regulation. A gradient freezing-based ice-templating method was employed to prepare the biopolymer-based scaffolds with optic nerve-mimicking oriented microstructures, which guided directional growth of PC12 cells. Notably, by harnessing the exceptional ROS-scavenging capability of PDA NPs, the GA@PDA nanocomposite scaffold could regulate the oxidative stress and inflammatory microenvironment and stimulate the polarization of retinal microglia/macrophages toward the M2 phenotype in the absence of exogenous biological components. The implantation of GA@PDA scaffold in an ONC rat model demonstrated enhanced efficacy in rescuing RGCs and promoting axon regeneration. Taken together, our study may provide new insights into the development of engineered biomaterials for optic nerve regeneration.

## Electronic supplementary material

Below is the link to the electronic supplementary material.

Supplementary Material 1: SEM images of scaffolds prepared with different concentrations, degradation curves of scaffolds, compressive stress-strain curve of the GA scaffold, SEM images of GA@PDA scaffold, Live/Dead staining of PC12 cells after co-incubation with scaffolds, surgical images, and macroscopic images of the optic nerves after different treatments.

## Data Availability

No datasets were generated or analysed during the current study.
