# Correction: implantation of biomimetic polydopamine nanocomposite scaffold promotes optic nerve regeneration through modulating inhibitory microenvironment

**DOI:** 10.1186/s12951-025-03862-5

**Published:** 2025-12-20

**Authors:** Tonghe Pan, Yate Huang, Jinfei Wei, Chen Lai, Yangjun Chen, Kaihui Nan, Wencan Wu

**Affiliations:** 1 State Key Laboratory of Ophthalmology, Optometry and Vision Science, School of Ophthalmology & Optometry, Eye Hospital, Wenzhou Medical University, Wenzhou, 325027 Zhejiang China; 2 National Engineering Research Center of Ophthalmology and Optometry, Institute of Biomedical Engineering, Eye Hospital, Wenzhou Medical University, Wenzhou, 325027 Zhejiang China; 3 Shenzhen Key Laboratory of Human Tissue Regeneration and Repair, PKU-HKUST ShenZhen- HongKong Institution, Shenzhen, 518057 Guangdong China; 4 National Clinical Research Center for Ocular Diseases, Eye Hospital, Wenzhou Medical University, Wenzhou, 325027 Zhejiang China; 5 Oujiang Laboratory (Zhejiang Lab for Regenerative Medicine, Vision and Brain Health), Wenzhou, 325000 Zhejiang China

**Correction: Journal of Nanobiotechnology (2024) 22:683.**

10.1186/s12951-024-02962-y

In this article, the author discovered an inadvertent error in Fig. 4.e, specifically in the second row of the M2 macrophage staining (CD206 and DAPI). The images for the LPS group and GA@PDA + LPS group appeared too similar, and upon examination of our original data, the author identified an error that occurred during the process of adjusting the fluorescence pseudo-color (from green to yellow). The incorrect image was used for the LPS group (the second row, first column in Fig. 4.e).

For completeness and transparency, the correct and incorrect versions of Fig. 4. are displayed below.

Incorrect Fig. 4.

**Fig. 4:**
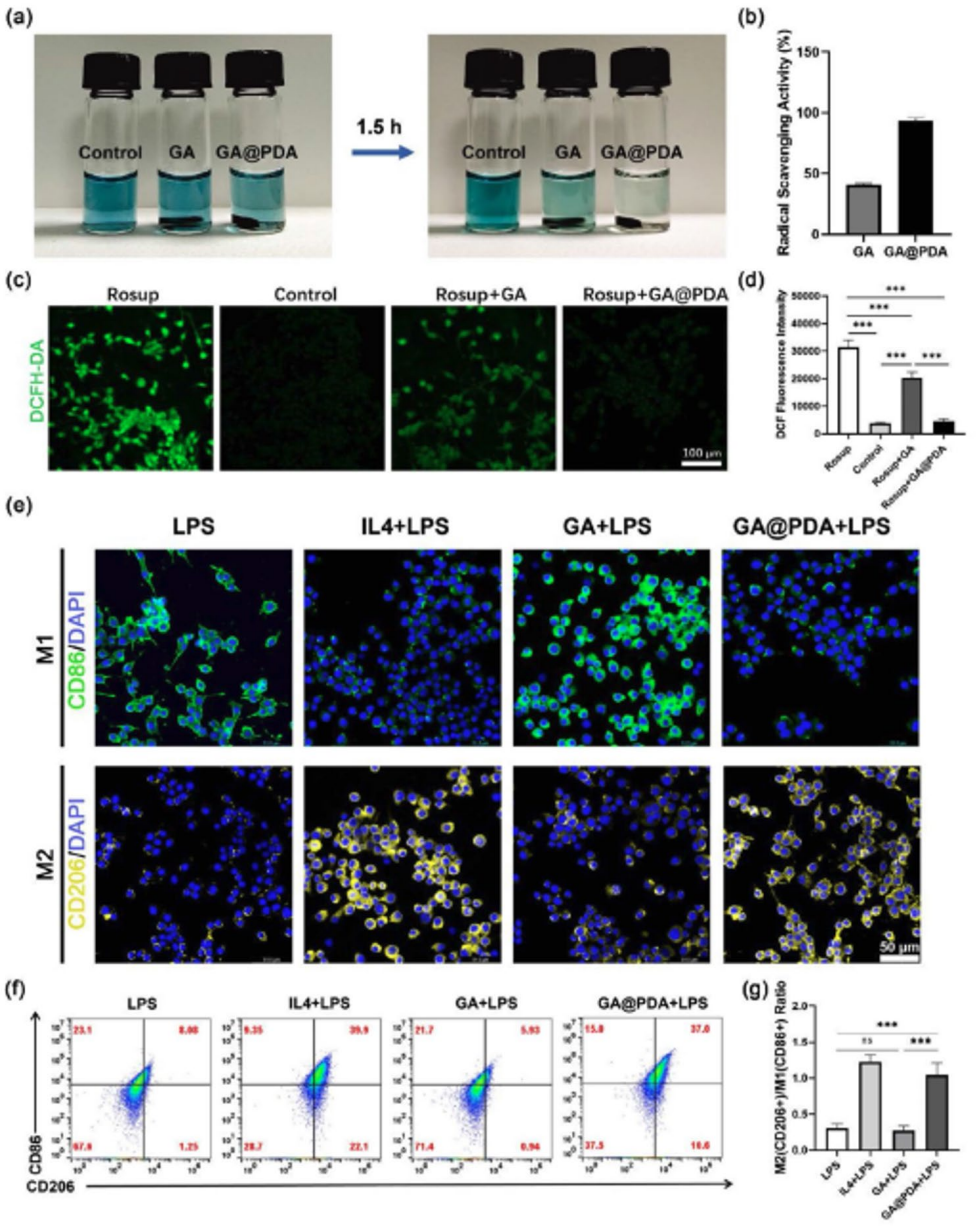
Evaluation of anti-oxidative and immune-regulating effects of GA and GA@PDA scaffolds. (**a**) The color change of ABTS⁺· solution after co-incubation with GA and GA@PDA scaffolds. (**b**) ABTS⁺· scavenging rate of the scaffolds. (**c**) DCF fluorescence images and (**d**) mean fluorescence intensity of PC12 cells upon various treatments (n = 5). (**e**) CD86 and CD206 staining images of RAW264.7 cells upon various treatments. (**f**) Representative flow plots of M1 (CD86+) and M2 (CD206+) of RAW264.7 cells upon various treatments. (**g**) Quantitative data of M2 (CD206+)/M1 (CD86+) ratio after different treatment (n = 3). ***p < 0.001, and ns, no significance

Correct Fig. 4.

**Fig. 4:**
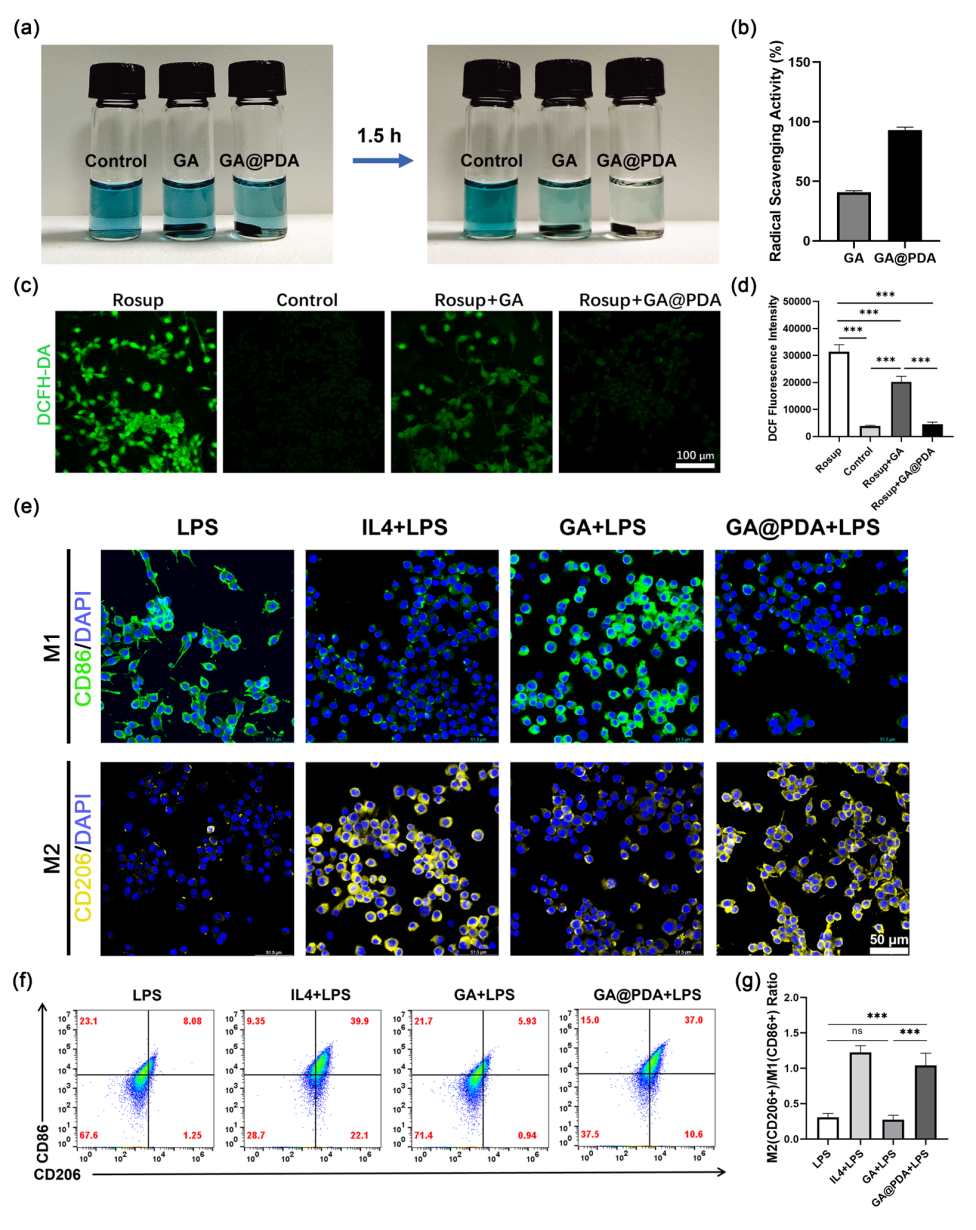
Evaluation of anti-oxidative and immune-regulating effects of GA and GA@PDA scaffolds. (**a**) The color change of ABTS⁺· solution after co-incubation with GA and GA@PDA scaffolds. (**b**) ABTS⁺· scavenging rate of the scaffolds. (**c**) DCF fluorescence images and (**d**) mean fluorescence intensity of PC12 cells upon various treatments (n = 5). (**e**) CD86 and CD206 staining images of RAW264.7 cells upon various treatments. (**f**) Representative flow plots of M1 (CD86+) and M2 (CD206+) of RAW264.7 cells upon various treatments. (**g**) Quantitative data of M2 (CD206+)/M1 (CD86+) ratio after different treatment (n = 3). ***p < 0.001, and ns, no significance

The original article has been corrected.

