# Supplementary material for: Implantation of biomimetic polydopamine nanocomposite scaffold promotes optic nerve regeneration through modulating inhibitory microenvironment
Source: J Nanobiotechnology. 2024 Nov 7;22:683. doi: 10.1186/s12951-024-02962-y (PMC11542345; doi:10.1186/s12951-024-02962-y)
Supplement: Supplementary file 1 — Supplementary Material 1: SEM images of scaffolds prepared with different concentrations, degradation curves of scaffolds, compressive stress-strain curve of the GA scaffold, SEM images of GA@PDA scaffold, Live/Dead staining of PC12 cells after co-incubation with scaffolds, surgical images, and macroscopic images of the optic nerves after different treatments. [file 12951_2024_2962_MOESM1_ESM.docx]

Supporting Information

# Implantation of Biomimetic Polydopamine Nanocomposite Scaffold Promotes Optic Nerve Regeneration through Modulating Inhibitory Microenvironment

Tonghe Pan^1,2,†^, Yate Huang^1,2,†^, Jinfei Wei^1^, Chen Lai^3^, Yangjun Chen^1,2,^*, Kaihui Nan^1,2,^*, Wencan Wu^1,4,5,^*

^1^State Key Laboratory of Ophthalmology, Optometry and Vision Science, School of Ophthalmology & Optometry, Eye Hospital, Wenzhou Medical University, Wenzhou, Zhejiang 325027, China

^2^National Engineering Research Center of Ophthalmology and Optometry, Institute of Biomedical Engineering, Eye Hospital, Wenzhou Medical University, Wenzhou, Zhejiang 325027, China

^3^Shenzhen Key Laboratory of Human Tissue Regeneration and Repair, PKU-HKUST ShenZhen-HongKong Institution, Shenzhen, Guangdong 518057, China

^4^National Clinical Research Center for Ocular Diseases, Eye Hospital, Wenzhou Medical University, Wenzhou, Zhejiang 325027, China

^5^Oujiang Laboratory (Zhejiang Lab for Regenerative Medicine, Vision and Brain Health), Wenzhou, Zhejiang 325000, China

^†^These authors contributed equally to this work.

*Corresponding author,

E-mail: chenyj@wmu.edu.cn (Y.C.); nankh@wmu.edu.cn (K.N.); wuwencan@wmu.edu.cn (W.W.)


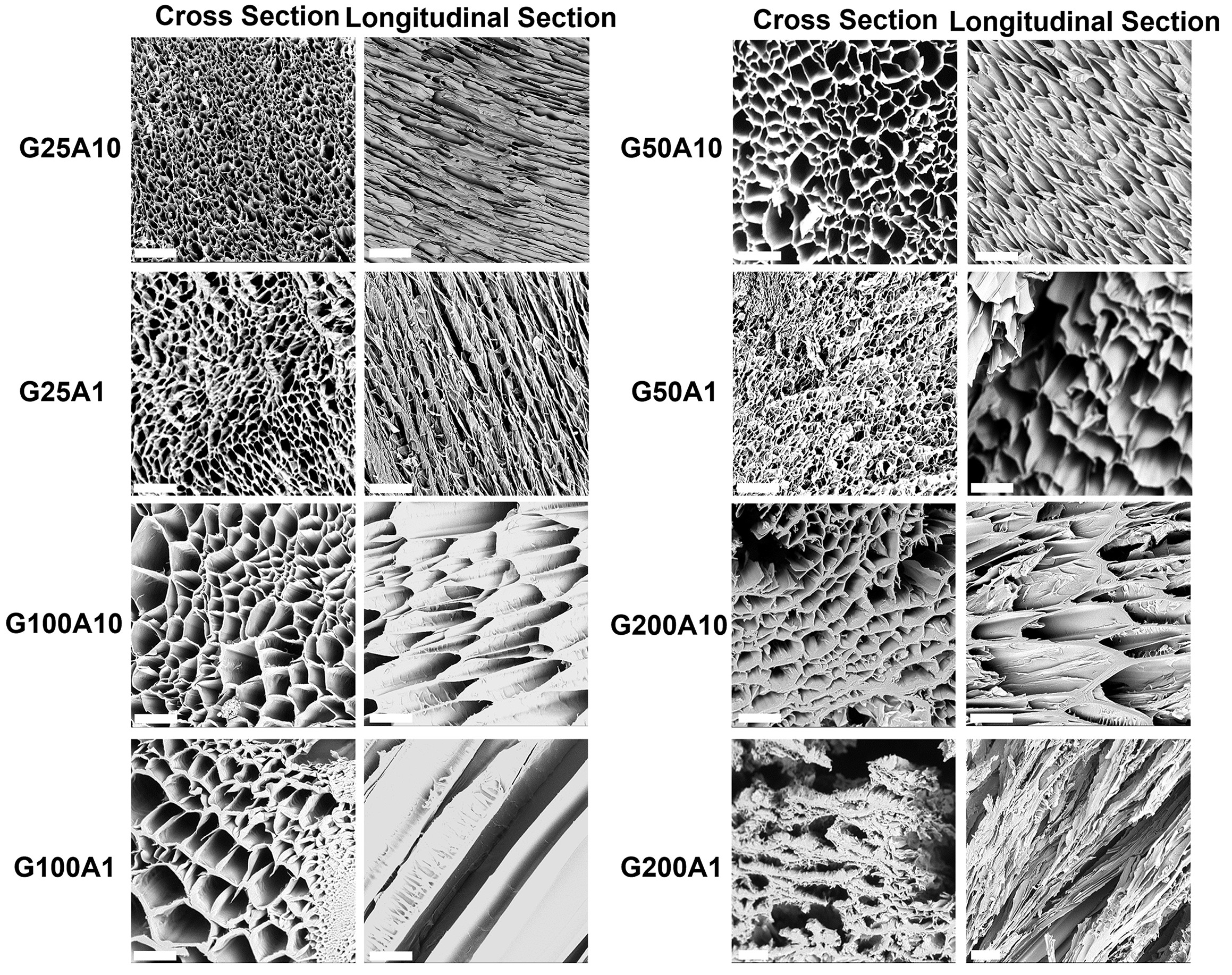


**Figure S1.** SEM images of cross-sectional and longitudinal views of scaffolds prepared with different concentrations of gelatin and sodium alginate. The concentrations of gelatin range from 25 to 200 mg/mL, while sodium alginate concentrations vary between 1 and 10 mg/mL. Scale bar = 100 μm.


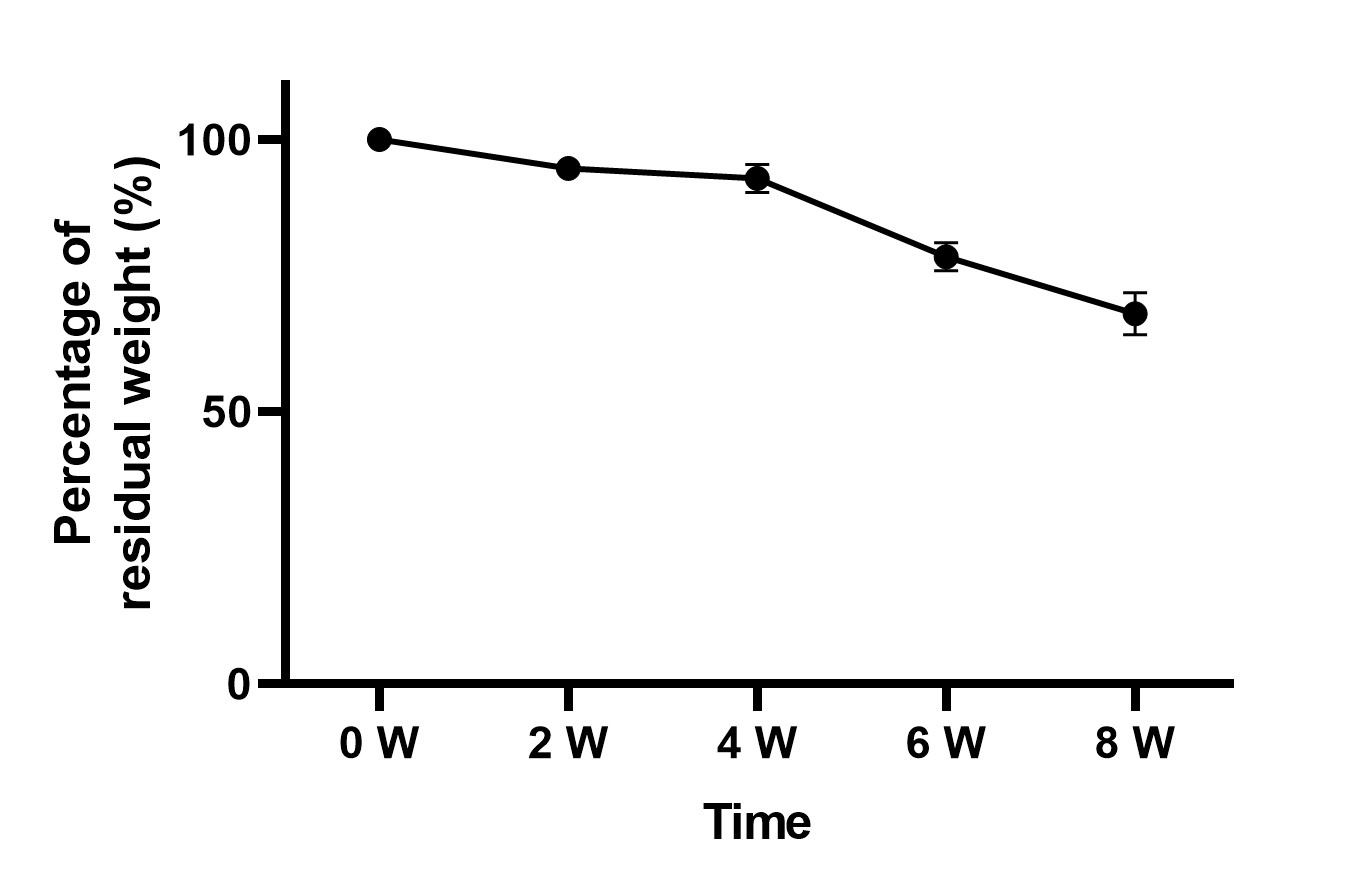


**Figure S2.** Degradation curves of scaffolds in PBS after cross-linking with genipin.


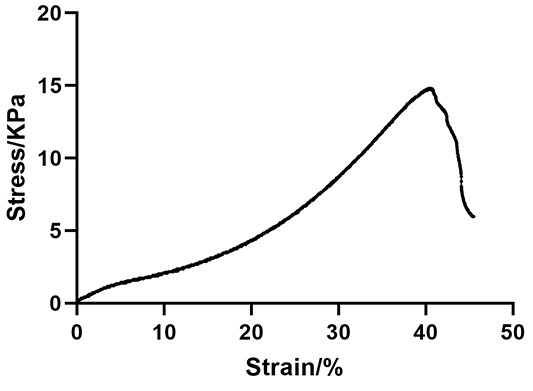


**Figure S3.** The compressive stress-strain curve of the GA scaffold cross-linked with genipin.


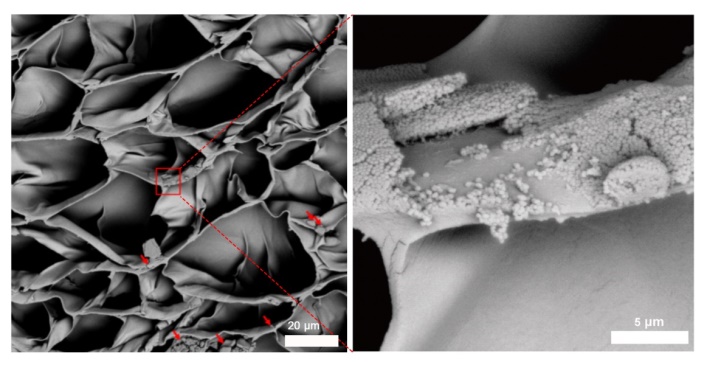


**Figure S4.** SEM images of GA@PDA scaffold loaded with polydopamine nanoparticles.


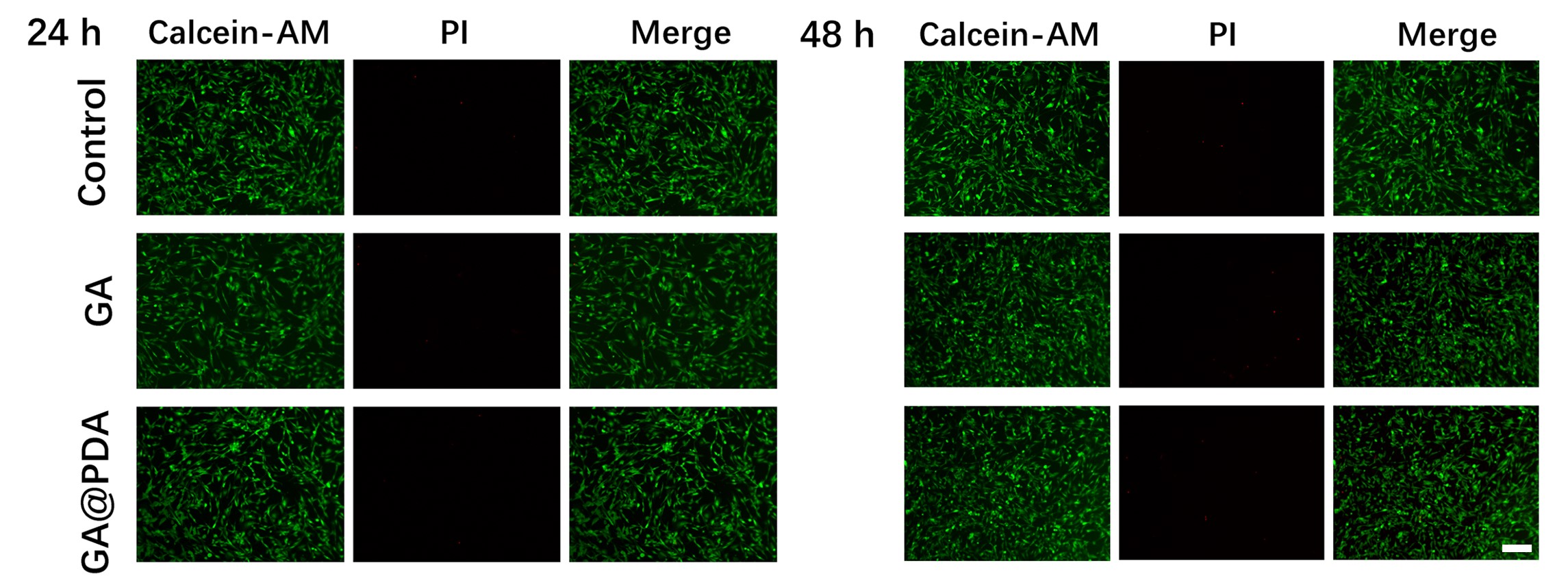


**Figure S5.** Dual-stained fluorescence images of PC12 cells after co-incubation with the extracts of GA and GA@PDA scaffolds for 24 h and 48 h via Live/Dead staining assay. Scale bar = 100 μm (n=5).


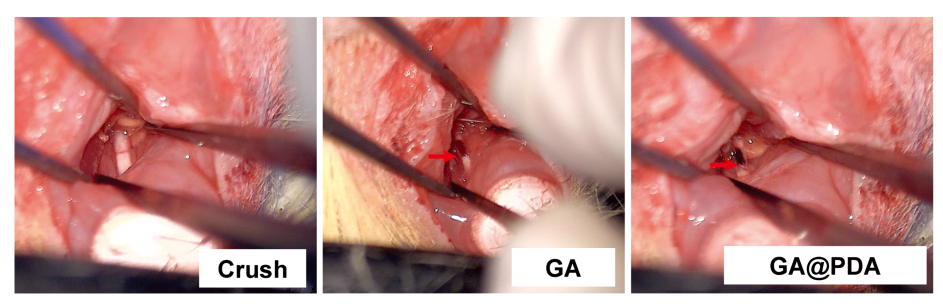


**Figure S6.** Surgical images in each group. Red arrows indicate the implanted scaffolds.


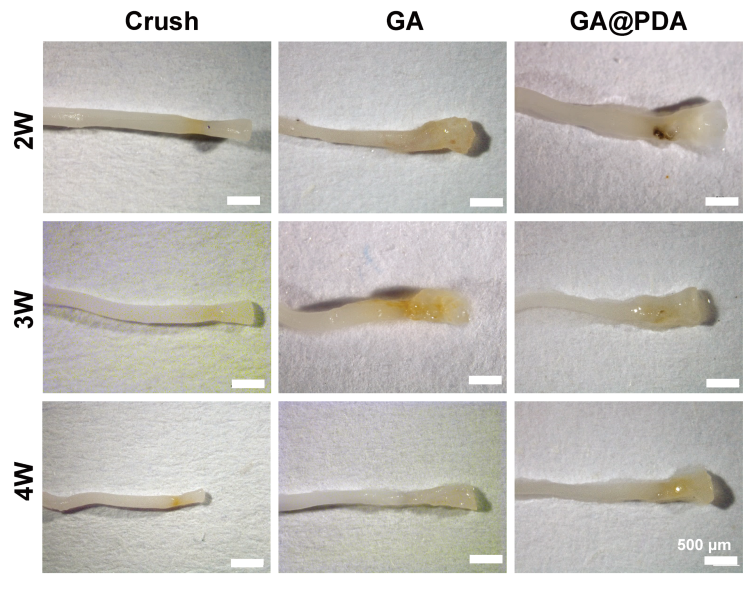


**Figure S7.** Macroscopic images of the optic nerves after different treatments for 2, 3, and 4 weeks, respectively.
